# 

**Published:** 1825-07-01

**Authors:** 


### MEDICO-CHIRURGICAL

### REVIEW.

*NEW SERIES.*

### VOLUME THREE.

[BEING VOL. VII. OF ANALYTICAL SERIES.]

CONDUCTED BY

ASSOCIATED PHYSICIANS AND SURGEONS;

AND SUPERINTENDED BY

JAMES JOHNSON, M.D.

MEMBER OF THE ROYAL COLLEGE OF PHYSICIANS OF LONDON,
AND PHYSICIAN EXTRAORDINARY TO HIS ROYAL HIGHNESS THE DUKE
OF CLARENCE.

Nec tibi quid liceat sed quid fecisse decebit

Occurrat mentemque domat respectus honesti.—CLAUD.

Surgeon-General's Office
LIBRARY
45744

LONDON:

*Printed by G. Hayden, Little College Street, Westminster.*

Published by Burgess & Hill, Windmill Street, Haymarket; Baldwin, Cradock, and Joy, Pater-Noster Row; Kingsbury, Parbury, & Allen, Leadenhall Street; Highley, and T. & G. Underwood, Fleet Street; Callow & Wilson, Princes Street, Soho; Cox & Son, Borough; Jackson, Borough; Anderson, West Smithfield; John Cox, Berners Street, Oxford Street; Adam Black, Edinburgh; D. Lizars, Princes Street, Edinburgh; Richard Griffin & Co. Glasgow; Hodges and M'Arthur, Dublin; Messrs. Treuttel & Wurtz, Paris and Strasburg.

1825.

### BIBLIOGRAPHICAL RECORD;

OR,

*Works received for Review between the 15th of March, 1825, and the 15th of June 1825.*

1. A Short Treatise on Operative Surgery, describing the principal operations as they are practised in England and France; designed for the use of students in operating on the dead body. By Charles Averill, M.R.C. M.C.S. and Surgeon to the Cheltenham Dispensary and Casualty Hospital, &c. &c. 2nd Edition, considerably enlarged, and with the addition of Drawings. London, 12mo. pp. 251. 4 plates, 1825.

---

2. Illustrations of the Arteries connected with Aneurism, and Surgical Operations. These Plates are intended to explain the relative Position of the Arteries, in respect to the surrounding Parts, and the Organs to be met with in such Operations, both external and internal to their Sheaths. No. 2, containing Plates II. III. and IV. of a Complete Series By G. D. Dermott, M.R.C.S. London, folio, 1825. Price Nine Shillings,

☞ *Whoever takes the trouble to examine these lithographic plates attentively, will agree with us that they are among the most correct illustrations of the subjects embraced which have ever been published. They do Mr. Dermott infinite credit.*

---

3. A Manual of Pharmacy. By William Thomas Brande, F.R.S. Secretary to the Royal Society of London; Fellow of the Royal Society of Edinburgh; Member of, and Professor of Chemistry in the Royal Institution of Great Britain; Professor of Chemistry and of Materia Medica to the Society of Apothecaries of the City of London; Honorary Member of the Literary and Philosophical Society of New York, of the Physico-Medical Society of Ertangen, of the Imperial Pharmaceutical Society of St. Petersburgh, of the Imperial Society of Naturalists of Moscow, &c. &c. London, 8vo. pp. 556. 1825.

4. Illustrations of Acoustic Surgery. By Thomas Buchanan, C.M. Licentiate of the University of Glasgow, Member of the Royal Medical Society, and Corresponding Member of the Phrenological Society of Edinburgh, Honorary Member of the Royal Jennerian Society, &c. &c. Surgeon to the Hull Dispensary for Diseases of the Eye and Ear, and author of the Guide to Acoustic Surgery. London, 8vo. pp. 118. Five Plates. 1825.

---

5. Manuel d'Anatomie, Generale, Descriptive, et Pathologique. Par I. F. Meckel, Professeur d'Anatomie à l'Université de Halle. Traduit de l'Allemand, par M. M. Jourdan et Breschet. 3 tomes. Paris, 1825.

☞ *This is the best System of Anatomy ever published. Here would be an excellent book to translate. It might be compressed into two closely printed volumes. It is certainly as much wanted in this country as in France; and is well deserving the speculation of the bookseller, leaving all other views out of the question.*

---

6. Instructions to Mothers and Nurses on the Management of Children in Health and Disease; comprehending Directions for regulating their Diet, Dress, Exercise, and Medicine; with a variety of Prescriptions adapted to the Use of the Nursery, and an Index of Medical Terms. By James Kennedy, M.D. Octavo, small, pp. 329. Glasgow and London, 1825.

☞ *This is by far the most scientific of any popular work on the Management of Children, as indeed might well be expected, from the known character of the author as a judicious and able practitioner—a sound pathologist—and a man of general science and literature. The work, though professedly addressed to Mothers and Nurses, is well deserving the attention of the junior branches of the Profession, who are often ill prepared—indeed we might say ill qualified, to answer the enquiries of parents respecting the management of their offspring. We, therefore, recommend this little volume to their library, as one that will probably be more useful to them than many of larger dimensions and bolder claims. We shall take an early opportunity of giving a farther account of it to our readers.*

---

7. The Carolina Journal of Medicine, Science, and Agriculture. Conducted by Drs. Simons and Michel. No. 1, for January, 1825. Pp. 100. Price 4 Dollars annually. Published quarterly.

☞ *We wish success to this first attempt to publish a medical journal in the State of South Carolina.*

---

8. Revue Medicale, Françoise et Etrangère, &c. Mois de Mars, 1825.

*In Exchange.*

---

9. An Essay on Tetanus, founded on Cases and Experiments. By Joseph Swan, Member of the Royal College of Surgeons, &c. &c. Octavo, pp. 98. Longmans, London, 1825.

---

10. Practical Remarks on Lacerations of the Uterus and Vagina: with Cases. By Thomas M'Keever, M.D. Late Assistant to the Dublin Lying-in-Hospital. Octavo, pp, 82. London 1824. Received 6th May, 1825.

---

11. A Physiological Essay on Digestion. By Nathan R. Smith, M.D. Professor of Anatomy and Physiology in the University of Vermont. Octavo, pp. 93. New York, 1825.

---

12. Outlines of Lectures on Mental Diseases. By Alex. Morison, M.D. of the Royal College of Physicians of London and of Edinburgh, &c. Octavo, pp. 72. Edinburgh, 1825.

---

13. The New York Medical and Physical Journal, (published quarterly.) Nos. 9, 10, 11, 12, and 13, to April, 1825. *(In Exchange.)*

☞ *This Journal, which is highly respectable, is now conducted by Dr. John B. Beck, Dr. Francis having retired.*

---

14. The Philadelphia Journal of the Medical and Physical Sciences. Nos. 17 and 18, for November, 1824, and February, 1825. *In Exchange.*

---

15. Observations on the Cholera Morbus of India: a Letter addressed to the Hon. the Court of Directors of the East India Company. By Whitelaw Ainslie, M.D. M.R.A.S. late of the Medical Staff of Southern India. Octavo, pp. 90. Kingsbury and Co. London, May, 1825.

☞ *We did not receive this little work till after the article on Cholera, in this number, was printed, otherwise it should have been included. We hope to be able to notice Dr. Ainslie's work next quarter.*

---

16. Further Observations on the Lateral or Serpentine Curvature of the Spine, and on the Treatment of Contracted Limbs; with an Enquiry into the Effects of Various Exercises, and other Means which are used to prevent or cure these Deformities: being a Supplement to the Work on Distortions of the Spine, and Bones of the Chest. By John Shaw, Surgeon, and Lecturer on Anatomy. Octavo, pp. 195. Several Woodcuts. Longman and Co. May, 1825. ☞ *In our next.*

---

17. Obstetric Studies: comprehending a Treatise on Parturition; likewise the various accompanying Symptoms during Pregnancy, and subsequently to Labour: with Descriptive References and Practical Observations. A Series of Plates is also published as a separate Work, in Two Classes. *Class* 1. Represents the Different Presentations, and Progressive Stages of Natural Labour, by a Peculiar Mechanical Process. *Class* 2. Contains a variety of Anatomical Subjects necessary to Elucidate the Obstetric Art. The Book and Plates together will furnish a Complete System of Practical Instructions upon the subject treated of, and it has hitherto been thought advisable to give references to the tables in this work, through a distinct publication, &c. By James Hogben, Surgeon. Quarto, pp. 170.

" 2. Anatomical Tables of the Human Gravid Uterus, in Two Classes: By the late Mr. Hogben, of Berners Street, Surgeon. Dedicated to the Royal College of Surgeons, London. The First Class consists of twenty-two Plates (large and small,) representing the different presentations of the Head of the *Fœtus*, (with its progressive stages, as at the time of *Labour*,) surrounded by the distended *Uterus*, showing the application of the *Forceps* and *Vectis*; exhibiting *Parturition* in a clear and comprehensive manner, by a peculiar mechanical process, which, although composed of paper, and laid perfectly flat, is yet contrived in such a manner that the real Forceps may be used, so as to give a true idea of its proper application on a living subject.

" The *Decidua*, and *Decidua Reflexa*, rendered singularly conspicuous, with a series of Anatomical Subjects essential to elucidate the Obstetric Science.

" The two Classes consist of nearly thirty Plates, with a descriptive Index to each Plate.

" This Work is supposed to bear a greater resemblance to Nature than has ever appeared before on paper; and the Author trusts it may be useful to the young Practitioner, as well as to the Student attending Lectures, and not altogether uninteresting to every Medical Man.

"Some eminent Professors of Midwifery having, in delivering *Lectures*, availed themselves of these TABLES, and been pleased to recommend them to their Pupils, this affords the best proof of the utility of the Work."

---

18. Observations on Extraction of Diseased Ovaria; Illustrated by Plates coloured after Nature. By JOHN LIZARS, Surgeon, Author of the System of Anatomical Plates. Folio, pp. 24, Five Coloured Plates. Edinburgh and London, May 1825. ☞ *In our next.*

---

19. A Popular Explanation of the Elements and General Laws of Chemistry. By WALTER WELDON. 8vo, pp. 622. London, June, 1825. Price 12s.

---

20. Parental Guide, during the Progress of Cow-Pock Disease. By JOSEPH MORLEY, Surgeon of Willingore, in the County of Lincoln, &c. 8vo, pp. 40, with a Coloured Plate.

---

21. A Letter to the Right Hon. W. Huskisson, M.P. President of the Board of Trade on the Quarantine Bill. By A. B. GRANVILLE, M.D. &c. 8vo, stitched, pp. 16.

---

22. Novus Thesaurus Semiotices Pathologiæ quem Collegit atque Edidit, MAURITIUS HASPER, M.D. Universit. Lit. Lipsiensi, &c. &c. 8vo, pp. 470. Leipsic, 1825.

☞ *We return our worthy friend Dr. Harper, our sincere thanks for this very excellent work, of which we shall take notice in our next article on the subject of Semiology.*

---

23. Geschichte der Augenheel Kunde in Sachsen. Von Dr. FRED. AUGUST. AMMON. 8vo, pp 72. Leipzig, 1824.

---

24. Parallele der Frangesischen und Deutcshen Chirurgie. Von Dr. FRED. AUGUST. AMMON. 8vo, pp. 483. Leipzig, 1825.

---

25. A Brief Sketch of the Progress of Opinion upon the Subject of Contagion; with some Remarks on Quarantine. By WILLIAM MACMICHAEL, M.D. F.R.S. &c. 8vo, Sewed, pp. 32. June, 1825.

---

26. Small Pox and Cow-Pox: comprehending a Concise History of those Diseases, &c. &c. By JOHN JENNINGS CRIBB, Member of the Royal College of Surgeons. 8vo, sewed, pp. 80. Cambridge and London, June, 1825.

☞ *The substance of this publication was lately read at the Medico-Chirurgical Society. We are very sorry to observe in it the death of a young gentleman (Mr. Barrett of Cambridge) by confluent small pox, after well defined vaccination.*

---

*** NOTE.—Authors and publishers will readily appreciate the importance of having their works recorded, with full length titles, on this list, which stands as a perpetual advertisement so long as the Journal lasts, and as far as it extends. The republication of the Journal in America enhances the advantages of the BIBLIOGRAPHICAL RECORD, on which no work can be entered, unless transmitted, free of expence, to the Editor, under sealed cover to the publishers, or in any other way most convenient to the parties concerned.

### *Additional Subscribers since last Quarter.*

A.

Austin, Joseph William, M.D. one of the Physicians to the Cork Dispensary, and Humane Society, Cork.

B.

Beattie, Dr.

C.

Campbell, John, Esq. Surgeon, Larges, N. Britain.
Carr, Mr. John, Surg. Montrose
Clinton, Dr.
Coxhead, Mr. John, Student.

D.

Daniel, Mr. Surgeon.
Davidson, Mr. Duke Street, Westminster.
Dawson, Mr. Surgeon.
Dixon, Mr. John, Surgeon, Ronald Kirk.

E.

English Medical Book Society, Paris.

F.

Fogarty, Dr.
Folds, Mr. Assist. Surgeon, R.N.
Fragon, Dr.

G.

Gray, Mr. Surgeon.
Greenhalgh, Dr. G. F. H. Bachelor of Medicine, Bury, Lancashire.
Gunning, John, Esq. Rue Foubourg, St. Honoré, No. 35, Paris.

H.

Harrold, E. Esq.
Hereford Medical Book-Society.
Heron, Dr.

J.

Johnson, Charles, Esq. Surgeon, Rotherhithe.
Johnston, Mr. Ebenezer, Surgeon, R.N.

K.

Kertletoot, Dr. J. L. Gand.
Kirwan, Dr. Dublin.
King, Gilbert, Esq. Surgeon, R.N.

M.

M'Mienn, Mr.
M'Naughty, John, Esq.
Montifiore, Dr. J. J. Surgeon, &c. Little Alie-st. Goodman's Fields.
Morrison, R. Esq. Surgeon in the Royal Navy, F.G.S. &c.
Mortefrine, J. J.

O.

Orpen, Dr. Charles.

P.

Perry, Mr. Henry, Member of the Royal College of Surgeons of London, Licentiate of the Society of Apothecaries, Henbury, near Bristol.

R.

Robinson, Mr. M.R.C.S. Huddersfield.
Ruddiman, Dr.

S.

Shaw, Mr. Surgeon, Alston, Cumberland.

T.

Thomson, T. Hale, Esq. New Cavendish Street.

V. & W.

Vereker, Dr. Dublin.
Walkins, Mr. J. P. Surgeon, Caermarthen.
Weir, Mr. Daniel, Greenock.
Whisher, Mr. H. Surgeon, Hastings, Sussex.
Williams, Henry, Esq. Surgeon, M.R.C.S. London, Glanconway.
Wishart, Mr. Robert, Student.
Wood, Mr. J. F. Lecturer on Chemistry at the Middlesex Hospital

---

*P. S.* The new College of Physicians opened on Friday, the 25th June, with great eclat. The inaugural discourse was pronounced by Sir Henry Halford. More particulars in our next.

